# Carbon stock estimation of mixed-age date palm (*Phoenix dactylifera* L.) farms in northeastern Ethiopia

**DOI:** 10.1016/j.heliyon.2022.e08844

**Published:** 2022-01-28

**Authors:** Mulugeta Betemariyam, Tamiru Kefalew

**Affiliations:** Madda Walabu University, P.O. Box 247, Robe, Ethiopia

**Keywords:** Biomass, Carbon stocks, Date palm, Farm, Ethiopia

## Abstract

The date palm (*Phoenix dactylifera*) is a fruit tree that grows from 392 to 1500 m above sea level. In addition to their socioeconomic, traditional, and religious value, it is a tree that tolerates high temperatures, drought, and salinity better than many other fruit crop plant species and plays an important role in the balancing and sequestration of atmospheric carbon. Date palm has been cultivated by agro pastoralists in Northeastern Ethiopia since ancient times, but no research has been done on the carbon stock of date palm farms (DPF) in the region. Therefore, the focus of the current study was to examine the carbon storage capacity in the biomass and soil of a DPF in the Aysaita and Afambo Districts of Northeastern Ethiopia. The ages of recorded date palm on the plot were classified into three age classes using information collected from the farm owners: 1st age class (for plantations less than 10 years), 2nd age class (for plantations between 10 and 20 years), and 3rd age class (for plantations older than 20 years). In the DPF, 45 main plots (20 m × 20 m) were established for tree species inventory. In the main plots, three 1 m × 1 m subplots were set up to collect soil samples. A total of 360 soil samples were collected; 180 for soil organic carbon fraction analysis and 180 for bulk density determination. The total carbon stock was calculated by adding the carbon stocks in biomass and soil (0–60 cm depth). Date palm trees accounted for 98.79% of total biomass carbon stocks in the date palm farm. The average aboveground biomass carbon stock of date palm trees older than 20 years was 1.55 and 1.36 times higher than the first and second age classes, respectively. Date palm trees between the ages of 5 and 20 years contributed 69.45% of total biomass carbon stocks (Mg C ha^−1^). Soil organic carbon made for 32.9% of total carbon stocks. Our research found that the date palm farm of this study would contribute to emission reduction and carbon sink enhancement, as well as improving local livelihoods in the study area.

## Highlights

• The highest numbers of date palm trees were found in the 10–20 age class among the available date palms.
• Date palm trees older than 20 years had a mean aboveground biomass carbon stock of 159.50 kg/plant.
• The SOC was accounted for 32.9% of total carbon stocks.
• The total carbon stock of the DPF was 82.3 Mg C ha^−1^.

## 1 Introduction

The date palm (*Phoenix dactylifera*) is a fruit tree that can be found in a wide range of geographic, soil, and climatic conditions around the world. It grows from 392 m below to 1500 m above sea level [1]. It tolerates high temperatures, drought and salinity more than many other fruit crop plant species [2]. It is a large tree with very dense leaves (nearly 4–5 m), and each leaf has approximately 150 leaflets (each leaflet is around 30 cm in length and 2 cm in width) [3]. Likewise, Date palm is a large deep rooted tree which its height ranges from 15 to 25 m. The amount of CO_2_ absorbed is, in fact, proportional to the size of the plant's green component; the absorption of CO_2_ and carbon stocks in the trunk and roots of Date palms are higher than those of other tree species [4].

In their investigation of carbon stocks between land uses, Sanquetta et al. [5] found that land planted with palms provided 40 Mg C ha^−1^, while pasture provided only 8 Mg C ha^−1^. Similarly, a study published by [6] in Northeast India found that oil palm plantations stored more carbon (3.7 Mg C ha^−1^ year^−1^) than adjacent shifting agriculture fallows. According to [6], date palms have a crucial role in the balancing and sequestration of atmospheric carbon, especially when planted in low-productivity areas or on degraded lands, in addition to their socio-economic, traditional, and religious value.

Date palm has been cultivated in Ethiopia since the 19th century by agro pastoralists in the Afar, Somali, Gambella, Dira Dawa, and Benishangul-Gumuz regions [7]. Date palm is grown along the Awash River in the districts of Afambo, Aysaita, and Gewane in the Afar region (study area). Because of their geographical location, the Afar people have historically had commercial links with the Arab world. According to the [8] report, the Afar areas and the Arab world have a 1400-year-old historical connection. The date palm planting in the region was also inherited from the Arabs, according to the report.

According to [2], agro pastoralists in the area collect 26–45 kg of Date palm fruit per tree per year on average. Date palm was grown in the area by using traditional farming practices which passed down from their parents and grandparents through time on less than one hectare of farm land which is low compared to other countries [2]. According to [3] research, individual farmers in several Sub-Sahelian African countries had more than tens of hectares covered in date palm farm (DPF).

Despite some research on the Date palm's socioeconomic, traditional, and nutritional roles [2, 7, 8, 9, 10], no research on the carbon stock of DPF in Afar region has been done. As a result, this study was carried out with the overall goal of examining the carbon accumulation potential in the biomass and soil of a DPF in the Aysaita and Afambo Districts of Afar region, which has implications for climate change mitigation. The study provides researchers and other stakeholders about the system's contribution in carbon stock stocks for climate change mitigation. It also contributes to date palm tree conservation and promotes the ecosystem benefits that date palm trees provide to local populations in addition to their socioeconomic value.

## 2 Materials and methods

### 2.1 Description of the study area

**Figure 1:**
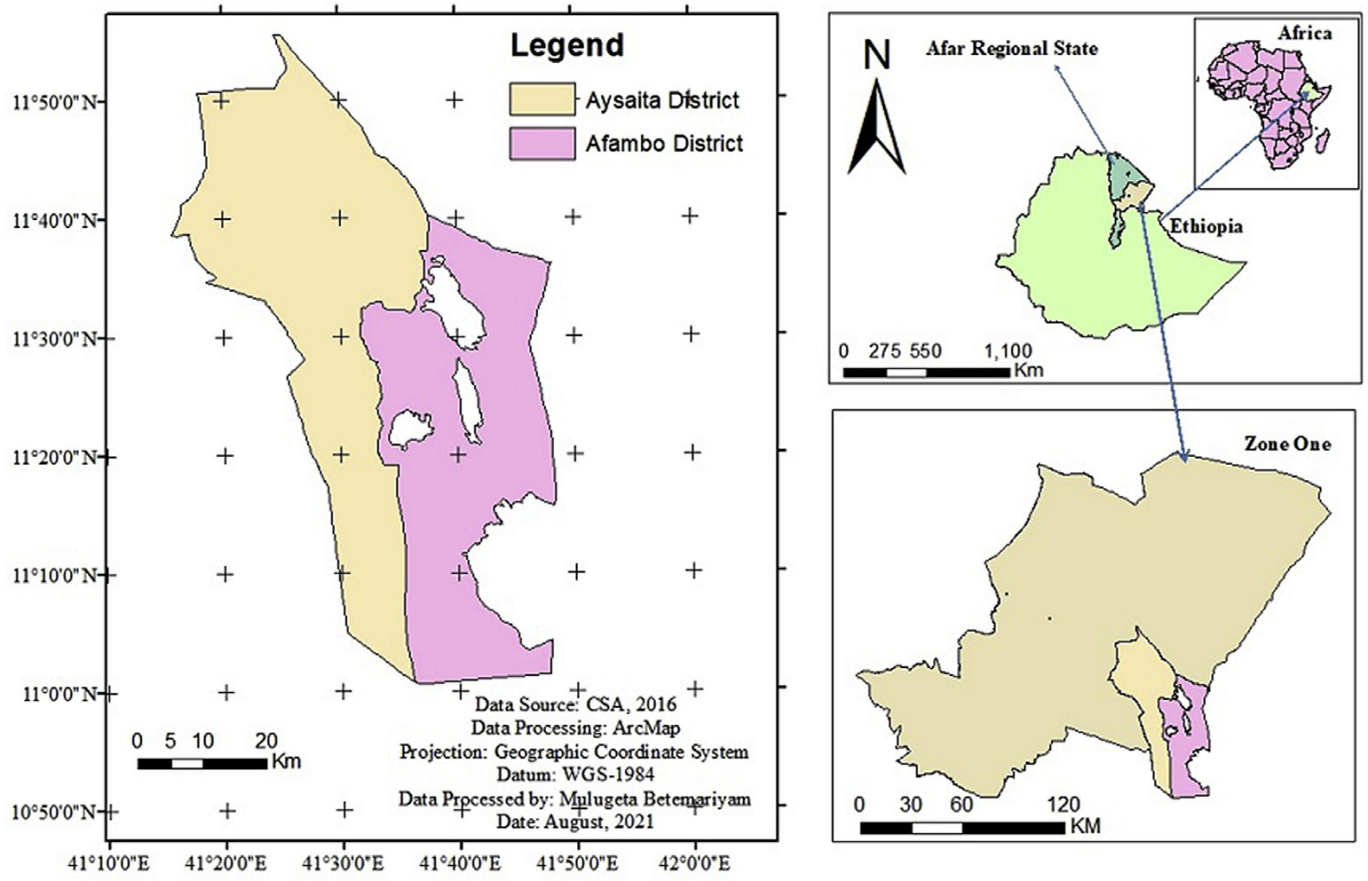
The map of Ethiopia showing Afar Regional State and the study area.

The research was carried out in Afambo and Aysaita districts of Afar Region Ethiopia (Figure 1). The districts are situated between 330 and 350 m above sea level. The area's average yearly rainfall is 122 mm, while the mean annual temperature is between 30 and 45 °C [2]. The first rains (sugum rains) fall between February and March, while the second rains fall between July and September (karma rains). The Awash River is the area's principal supply of water for DPF. *Prosopis Juliflora* is a woody plant found on the research area's farms in combination with date palm trees.

**Figure 2:**
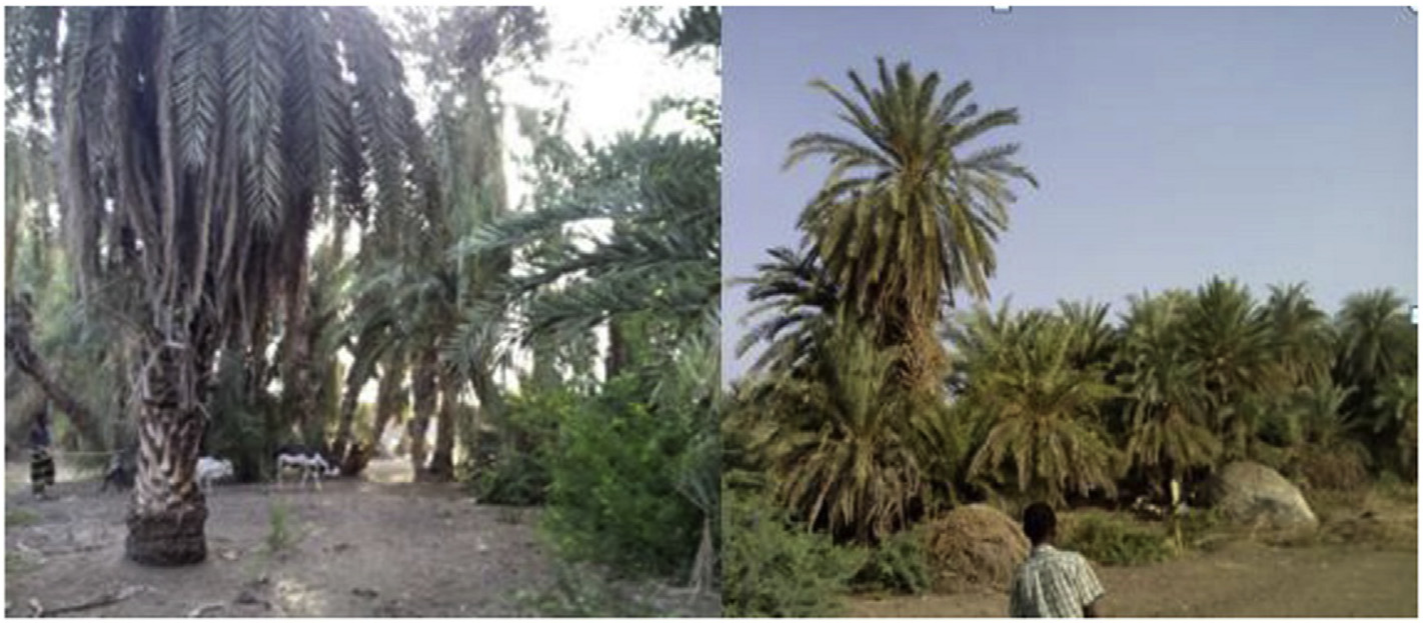
Photo of Date Palm farm in Aysaita District, Northeastern Ethiopia (Photo: Betemariyam M.).

In the study area, both local and improved date palm varieties were cultivated. Local varieties were produced by the majority of smallholder agro pastoralists. There are a few households that have improved date palm varieties on their farms. The majority of improved DPF in the research area are owned by the Afar pastoral and agro-pastoral research institute and the Afar bureau of pastoral agriculture development. For this study, all plots were established on DPF where smallholder agro pastoralists practiced by traditional farming methods (local varieties) (Figure 2).

### 2.2 Sampling design

From four districts with an experience of date palm production in Afar region, Afambo and Aysaita districts were purposively selected due to they have long year experiences on the production of date palm tree species. The majority of DPF owners have a farming experience of 15–40 years. Three kebeles, namely Lasabolo, Humadoyta and Berga were also purposively selected. The agro-ecology and climatology of selected kebeles were more or less categorized under similar range. According to report of [11], age is one of the determinant factors that influence the biomass of the palm and its structural measurements.

**Figure 3:**
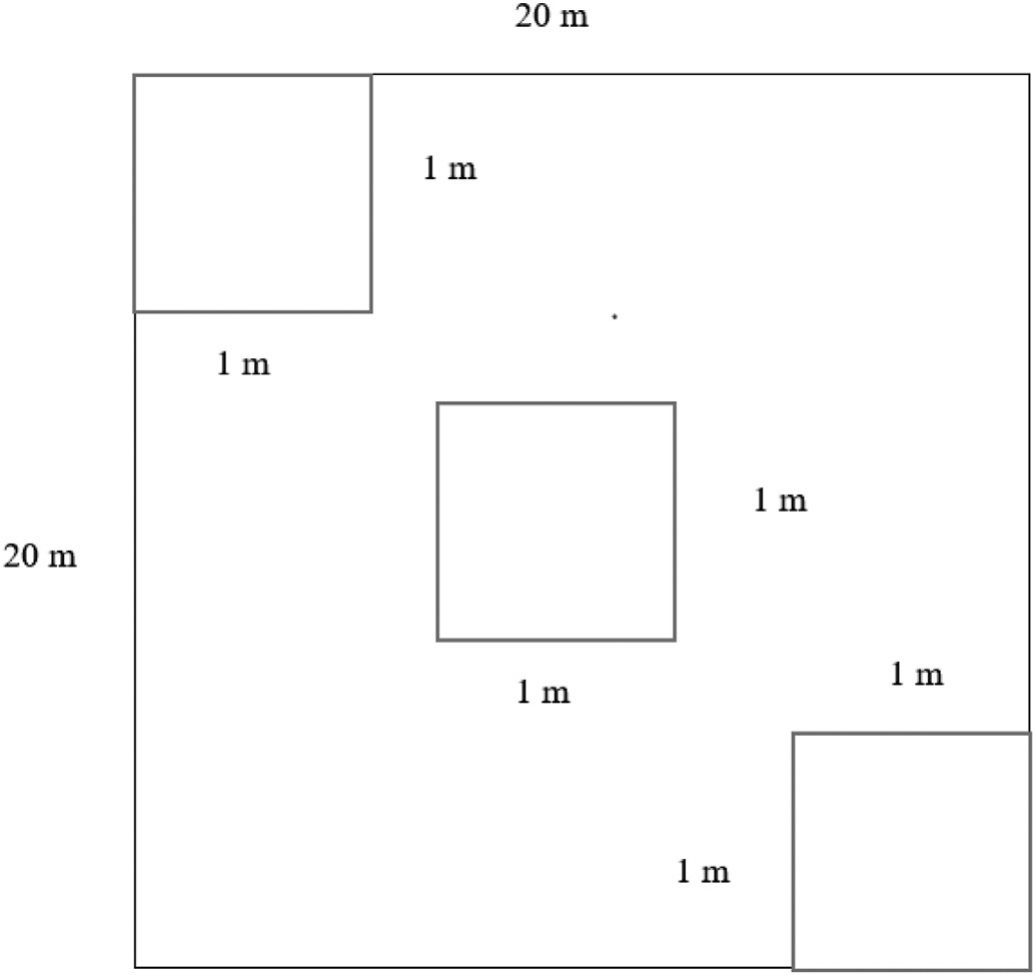
Plot size and design for field measurements.

Many studies has been undertaken and published on the estimation of palm biomass at various ages [4, 11, 12, 13, 14, 15, 16]. Similar approach was used to estimate date palm biomass in the current investigation. Using information obtained from the farm owners, the age of recorded date palm in the plot was categorized into three different age classes: 1st age class (for plantations less than 10 years), 2nd age class (for plantations between 10 and 20 years), and 3rd age class (for plantations older than 20 years). On DPF, a total of 45 nested plots measuring 20 m × 20 m with three 1 m × 1 m sub-plots were developed for species inventory and soil sampling (Figure 3).

### 2.3 Field measurements

In February 2018, field measurement was carried out. Using their standard instruments, each date palm's diameter at breast height (DBH) in centimeters, crown diameter (CD) in meters, and trunk height (Ht) in meters was measured. Each stem was measured at 30cm height from the ground in the case of multi-stemmed *Prosopis Juliflora* plants (more than 2 stems per plant). The sphere equation was used to compute the crown area (CA) of each date palm under each age class [4].

(1) $$\mathrm{CA}=\frac{\pi CD^{2}}{4}$$

### 2.4 Soil sampling

Soil samples were taken from the three sub-plots set up at the main plot's diagonal [17]. Soil samples were collected to determine the organic carbon percentage (%C), texture, and bulk density of the soil. Soil samples were taken from the 0–15, 15–30, 30–45, and 45–60 cm strata. A total of 360 soil samples were collected for lab testing.

### 2.5 Biomass and carbon content determination

[4] and [18] established specific allometric equations were used for calculating aboveground biomass (AGB) of date palm and *Prosopis juliflora*, respectively. Based on root to shoot ratios, belowground biomass (BGB) for both date palm and *Prosopis juliflora* was calculated. For conversion, a 58% and 50% C content was used for date palm and *Prosopis juliflora*, respectively [4, 18].

(2) AGBfordatepalm=CrownBiomass(CB)+TrunkBiomass(TB)

(3) $$CB=14.034e^{0.0554*\mathrm{CA}};\;R^{2}=0.8354$$

(4) $$TB=40.725Ht^{0.9719}\;;\;R^{2}=0.828$$

(5) BGBofdatepalm=AGB∗0.496(20%)

(6) $$\mathrm{AGB}\;\mathrm{of}\;\mathrm{one}\;\mathrm{and}\;\mathrm{three}\;\mathrm{stemed}\;\mathrm{Prosopis}\;\mathrm{juliflora}=\beta 0*BD^{\beta 1};\;R^{2}=0.8\;\mathrm{and}\;0.73\;\mathrm{respectively}$$

(7) $$\mathrm{AGB}\;\mathrm{of}\;\mathrm{two}\;\mathrm{stemed}\;\mathrm{Prosopis}\;\mathrm{juliflora}=\beta \text{0}+\left(\beta 1*\mathrm{BD}\right)+\left(\beta 2*BD^{2}\right)+\left(\beta 3*BD^{3};\;R^{2}=0.98\right)$$

Where, BD is the basal diameter measured at a height of 30cm from the ground and ß0, ß1, ß2 and ß3 are coefficients

(8) BGB=AGB∗0.2(20%)

### 2.6 Soil analyses

Soil analyses were undertaken at Wondo Genet College of Forestry and Natural Resources soil laboratory. For bulk density, soil samples were oven-dried for 24 h at 105 °C and weighed [19]. The core method was used to calculate bulk density [20]. The soil samples for %C were air dried and analyzed using Walkley and Black method [21]. Carbon fraction (percent) ∗ bulk density (g/cm^3^) ∗ layer thickness were used to determine SOC stocks (Mg C ha^−1^) (cm). Summing biomass carbon stocks (above-and-below) and SOC stocks yielded the total DPF carbon stock.

### 2.7 Data analysis

The normality of the data was check using the Kolmogorov-Smirnov test before further statistical analysis. Because there were different numbers of date palm trees at different ages, Welch's one-way ANOVA test was used to see if there was a statistical difference in the biomass carbon stocks among date palm age class. The Games-Howell test was used to compare means that exhibited significant differences at the 5% probability level. ANOVA was also used to see if there was a difference in mean SOC stocks as soil depth increased. Fisher's Least Significant Difference (LSD) post hoc test was used to compare means that demonstrated significant variations in SOC among four levels of soil depth. An independent sample t-test was used to determine whether there was a significant difference between total biomass and SOC.

## 3 Results

### 3.1 Characteristics of DPF

The majority of individual agro pastoralists in the research area held less than one hectare of date palm covered land. In most agro pastoralist farms, there are less than 30 date palm trees. *Prosopis juliflora* is a woody plant that grows alongside date palm trees. The highest numbers of date palm trees were found in the 10–20 age class among the available date palms (Table 1). Most household farms have less than 6 m × 4 m space between date palm trees.

**Table 1:** Components of DPF in Afambo and Aysaita districts, northeastern Ethiopia.

Component	Date palm in age class			*Prosopis juliflora*		
	5–10 years	10–20 years	Older than 20 years	One stemed	Two stemed	three stemed
N	139	272	117	26	34	56
DBH (cm)	27.03 ± 14.75	28.35 ± 13.18	35.56 ± 7.99	–	–	–
BD (m)	–	–	–	4.77 ± 1.9	4.73 ± 1.32	4.71 ± 1.42
H (m)	6.47 ± 1.21	7.42 ± 1	8.03 ± 1.34	3,27 ± 1.23	3.01 ± 1.97	2.97 ± 1.36
Ht (m)	3.74 ± 0.95	4.46 ± 1.02	4.71 ± 0.71	–	–	–
CD (m)	3.7 ± 1.16	4.15 ± 0.94	6.11 ± 1.1	–	–	–
CA (m^2^)	14.96 ± 10	14.22 ± 5.87	30.31 ± 10.9	–	–	–

Where: N is total number of trees recorded from total number of plots (45 plots); DBH is diameter at breast height; BD is basal diameter at 30 cm; H is total height; Ht is truck height; CD is crown diameter; and CA is crown area.

### 3.2 Biomass carbon stocks

**Figure 4:**
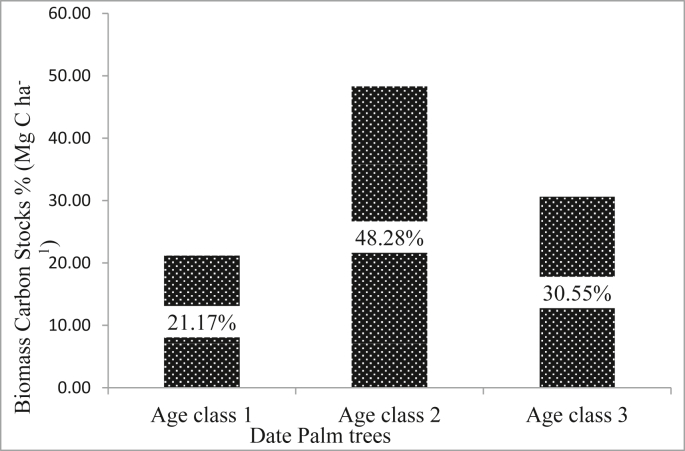
The percentage of biomass carbon stocks to the age of date palm trees in date palm farm.

The third age class (older than 20 years) of date palm trees in the study area had significantly higher aboveground biomass carbon stock (kg/plant) than the left age classes (Table 2). Date palm trees older than 20 years had a mean aboveground biomass carbon stock of 159.50 kg/plant that was 1.55 and 1.36 times larger than the first and second age classes, respectively. Date palm trees constitute 98.79% of the total aboveground biomass carbon stocks (Mg C ha^−1^) in DPF. From this, date palm trees between the ages of 5 and 20 years contributed 69.45% of total biomass carbon stocks (Mg C ha^−1^) on DPF (Figure 4).

**Table 2:** (Mean ± SE) Of the total carbon stocks in the DPF and three age classes of date palm trees in Aysaita and Afambo districts, Afar Region Ethiopia.

Biomass component	Date palm farms (Mg C ha^−1^)	Date palm trees per age class (Kg/plant)			Date palm trees (Mg C ha^−1^)	*Prosopis juliflora* (Mg C ha^−1^)
		1st age class	2nd age class	3rd age class		
AGBC	36.94 ± 1.84	102.81 ± 2.02	117.21 ± 1.38	159.50 ± 5.72	36.38 ± 1.84	0.56 ± 0.06
BGBC	18.21 ± 0.91	50.99 ± 1.01	58.13 ± 0.69	79.11 ± 2.83	18.10 ± 0.91	0.11 ± 0.01
TBC	55.15 ± 2.75	153.80 ± 3.03^a^	175.34 ± 2.07^b^	238.61 ± 8.55^c^	54.48 ± 2.75	0.67 ± 0.07

Between biomass component mean followed by different letters are significantly different and mean followed by similar letters are not significantly different at (p < 0.05).

The percentage of biomass carbon in date palm trees (Mg C ha^−1^) to total biomass carbon in DPF (Mg C ha^−1^) was 21.17%, 48.28%, and 30.55%, respectively, for ages 5–10, 10–20, and older than 20 years. *Prosopis juliflora* trees accounted for 1.21% of the system's total biomass carbon stocks.

AGBC is Aboveground Biomass Carbon; BGBC is Belowground Biomass Carbon and TBC is Total Biomass Carbon; 1st age class is date palm trees between 5-10 ages; 2nd age class is date palm trees between 10-20 ages; 3rd age class is date palm trees older than 20 ages.

### 3.3 Soil organic carbon (SOC) stocks

The overall mean value of SOC in the DPF was significantly higher (p < 0.05) at depths of 0–15cm and 15–30 cm than at 30–45cm and 45–60 cm (Table 3). The top two layers in a DPF accounted for 37.41% and 27.38% of the total SOC, respectively, with the remaining percent held by the subsequent layers. SOC stocks were nearly 22.77% and 12.75% higher at the upper two levels (0–15cm and 15–30cm, respectively) than at the lowest depth (45–60 cm).

**Table 3:** (Mean ± SE) Of soil organic carbon (SOC) in DPF in Aysaita and Afambo districts, Afar Region Ethiopia.

Variables	Depth (cm)	SOC (Mg C ha^−1^) (n = 45)
SOC Mg C ha^−1^	0–15 cm	10.16 ± 0.45^d^
	15–30 cm	7.44 ± 0.33^c^
	30–45 cm	5.58 ± 0.30^b^
	45–60 cm	3.97 ± 0.23^a^
	Total (0–60 cm)	27.15 ± 0.73

n number of sample plots. Between soil depth mean followed by different letters are significantly different (p < 0.05).

### 3.4 Total carbon stocks of DPF

The total carbon stock of the DPF was 82.3 Mg C ha^−1^. The SOC was accounted for 32.9% of total carbon stocks (Table 4). The percentage contribution of biomass carbon to total carbon stock in DPF was significant, accounting for about 67.1% of total carbon stock.

**Table 4:** Total carbon stocks of date DPF.

Carbon stocks	DPF (n = 20)
Total biomass	55.15 ± 2.73^b^
SOC 0–60 cm	27.15 ± 0.73^a^
DPF total	82.30 ± 3.46

Mean followed by different letters are significantly different and mean followed by similar letters are not significantly different at (p < 0.05).

## 4 Discussion

The Ht and CD of date palm trees between the ages of 5 and 10 are smaller than those of date palm trees between the ages of 10 and 20, as well as those older than 20 years. The Ht and CD of date palm trees increased as they grew older. The Ht and CD values of date palm trees older than 20 years were 1.26 and 1.65 times greater than those of date palm trees in 1st age class, respectively. Similarly, date palm trees between the ages of 10 and 20 had Ht and CD values that were 1.2 and 1.12 times higher than those of date palm trees in 1st age class, respectively. In line with this study, Issa et al. [22] found that Ht and CD are structural variables that are strongly and positively correlated with date palm tree ages.

It was observed that date palm trees of various ages store varying amounts of carbon in their biomass. In this study's DPF, date palm trees between the ages of 10 and 20 years stored much more carbon (kg/plant) in their biomass than date palm trees between the ages of 5 and 10 and older than 20 years. The Ht and CD of date palm trees could be the cause of this disparity. According to [22], the trunk, crown coverage, and total biomass of date palms increase in proportion to their age. The mean biomass carbon stocks of date palm trees older than 20 years in this study (238.61 kg/plant) were relatively greater than the mean biomass carbon stocks of date palm trees of the same age in Abu Dhabi, UAE (225.58 kg/plant) [4]. This discrepancy could be attributed to differences in Ht, CD and site management practices. Variation in site, palm varieties, ages, and farm management approaches, according to various assessments, has an impact on total biomass carbon stock [22, 23, 24]. According to an informal discussion with farm owners conducted during data collection, the producers' management approaches are traditional and have been passed down through their families. The responsible stakeholders who are required for the effective production of date palm trees have not provided any training sessions or extension services on date palm agronomic, management, or postharvest handling methods.

According to [25], the aboveground carbon stock of four, eight, and fifteen year oil palm farms in Tamil Nadu Regimes, India, was 20.44 kg/palm or 2.92 Mg C ha^−1^, 174.38 kg/palm or 24.94 Mg C ha^−1^, and 440.09 kg/palm or 62.93 Mg C ha^−1^, respectively. Aboveground carbon stocks for oil palm plantations range from 31 to 62 Mg C ha^−1^ for young cultivations of 10 years–96–101 Mg C ha^−1^ for stands of 14 and 19 years [26]. In Sumatra, Indonesia, aboveground carbon stores reach 9.2 Mg C ha^−1^ in 3-year plantations, and 35.4, 41.7, and 55.3 Mg C ha^−1^ in 10-, 20-, and 30-year plantations, respectively [27]. For the aboveground biomass, the authors estimated carbon stocks of 7.94, 18.11, and 10.51 Mg C ha^−1^ in 1st age class, 2nd age class, and 3rd age class of date palm trees in DPF, respectively.

DPF's total biomass carbon stock ranged from 28.73 to 109.77 Mg C ha^−1^. Palm trees dominated the DPF in the research area. In the DPF, date palm trees stored more than 98% of the biomass carbon. *Prosopis juliflora* makes a small contribution to the overall biomass carbon stock in DPF.

The mean biomass carbon stocks of DPF of this study is relatively higher than the biomass carbon stocks in homegarden (7.79 Mg C ha^−1^), parkland (7.79 Mg C ha^−1^), woodlot (31.12 Mg C ha^−1^) and boundary plantation (4.03 Mg C ha^−1^) in lowland, midland and highlands of Tigray region, northern Ethiopia [28]. The DPF of this study also stores higher biomass carbon stocks than biomass carbon stocks of oil palm (35 Mg C ha^−1^) in northeastern Brazil [5].

SOC is important to the global carbon cycle because it forms enormous carbon pools with long residence durations [29]. This study's total SOC at soil depth (0–60 cm) (27.15 Mg C ha^−1^) was within the range of soil organic carbon stocks reported for farmland worldwide (30–300 Mg C ha^−1^) [30]. The SOC stocks of date palm farm in the study area ranged between 17.48 and 40.01 Mg C ha^−1^ which are noticeably lower compared to the SOC stocks of other ecosystems soils. Tropical forest, tropical savannah, and tropical agricultural land had SOC stocks of 121–123 Mg C ha^−1^, 110–117 Mg C ha^−1^, and 80–103 Mg C ha^−1^, respectively, at the 60 cm layer [31].

The main land use type in the study region is livestock grazing, which is also the principal source of income for smallholder agro pastoralists. Grazing is recognized to have a significant impact on the structure, composition, and functioning of dryland ecosystems, including their capacity to fix and retain soil organic matter. According to [32, 33, 34, 35], changes in soil organic matter stocks with grazing are mostly a result of its effects on net primary production. Even while cattle may return a significant amount of nutrients and organic matter in the form of feces and urine, grazing reduces aboveground plant biomass significantly. Bardgett and Wardle [36] and Eldridge et al. [33] observed that grazing reduced plant biomass by 40% and litter cover by 25% on Australian rangelands, with more substantial effects in dry conditions and noticeable effects at moderate grazing intensities. Grazing of cattle increases soil compaction via trampling, reducing water infiltration and availability to plants while increasing runoff and soil erosion, further adding to soil organic matter losses [33, 34, 35]. Intensive grazing has also been observed to deplete soil organic matter stocks by trampling and burying biological soil crusts, lowering cover, biomass, and variety [37].

However, semi-arid crop production in Northern Ethiopia stores 25.8 Mg C ha^−1^ [38], which is equivalent to the current study. Semi-arid Acacia woodland in Southern Ethiopia stores 42.9 Mg C ha^−1^ [39], which is higher than the current study farmland. According to numerous researches, SOC demonstrates variation due to differences in land use regimes. This was mostly due to the availability of organic matter, the loss of SOM stability caused by tillage, and the subsequent mineralization of SOM aided by increased soil temperature and aeration [40].

## 5 Conclusions

DPF, according to the study, reduce emissions and improve sinks on the agricultural landscape while also supporting local livelihoods. The increments of biomass and soil organic carbon stocks of date palm farm was determined by the age of date palm trees, stem density, Crown diameter, and trunk height. Date palm trees that are older than ten years old, in particular, contribute significantly to the increase in biomass carbon in the date palm farm. *Prosopis juliflora* contributes less than 5% of biomass carbon stocks to the system. The biomass components of the date palm farm held the most carbon, showing that the trunks of date palm trees absorb more CO_2_ and store carbon.

## Declarations

### Author contribution statement

Mulugeta Betemariyam: Conceived and designed the experiments; Performed the experiments; Analyzed and interpreted the data; Contributed reagents, materials, analysis tools or data; Wrote the paper.

Tamiru Kefalew: Analyzed and interpreted the data; Wrote the paper.

### Funding statement

This work was supported by the 10.13039/501100015611Samara University.

### Data availability statement

Data will be made available on request.

### Declaration of interests statement

The authors declare no conflict of interest.

### Additional information

No additional information is available for this paper.
